# Allographic Agraphia for Single letters

**DOI:** 10.3233/BEN-2012-119008

**Published:** 2012-03-19

**Authors:** Alina Menichelli, Francesca Machetta, Antonella Zadini, Carlo Semenza

**Affiliations:** ^1^S.C. Medicina RiabilitativaOspedali Riuniti di TriesteTriesteItaly; ^2^Department of PsychologyUniversity of TriesteTriesteItaly; ^3^Department of NeuroscienceUniversity of Padovaand I.R.C.C.S. Ospedale S. CamilloLido di VeneziaItaly

**Keywords:** Allographic agraphia, word/letter dissociation, letter production, letter font, letter case

## Abstract

The case is reported of a patient (PS) who, following acute encephalitis with residual occipito-temporal damage, showed a selective deficit in writing cursive letters in isolation, but no difficulty to write cursive-case words and non-words. Notably, he was able to recognize the same allographs he could not write and to produce both single letters and words in print. In addition to this selective single letter writing difficulty, the patient demonstrated an inability to correctly perform a series of imagery tasks for cursive letters.

PS’s performance may indicate that single letter production requires explicit imagery. Explicit imagery may not be required, instead, when letters have to be produced in the context of a word: letter production in this case may rely on implicit retrieval of well learned scripts in a procedural way.

## 1. Introduction

Cognitive models of spelling and writing [7,8,15,16] distinguish between central processes ("sublexical", or "phonological", and "lexical" routes, graphemic buffer) involved in the generation of spelling, whatever the modality of output (handwriting, oral spelling, typing, writing with block-letters, etc.), and peripheral processes which are specific to one particular output modality. Three successive planning stages are described for handwriting. In the first stage, referred to as the "allographic system", the selection of the allograph, the appropriate font and case of the letter to be written, takes place. In the second stage, the graphic-motor pattern specifies the direction, relative size, position and order of strokes. The final stage, the graphic code, corresponds to the execution and control of neuromuscular commands.

Neuropsychological literature supports the psychological and neural reality of the allographic stage. A complex pattern of dissociations has emerged, providing useful insights into the functional organization of writing at this level. In fact, a wide range of selective deficits to specific allographic representations and functions has been reported. Some individuals show a disturbance in their ability to control the case of words, manifested in their writing in mIXeD CasES [5,10]. This phenomenon is thought to reveal the existence of format-independent abstract grapheme representations, that then need to be coded in a specific allographic form. Other patients have been described, instead, who are selectively unable to write in a single specific font or case [12,18,19,24,26]. These cases indicate that both the information regarding letter forms in different fonts and cases and the procedures responsible for accessing them (see [10,24] for a distinction) are represented with sufficient neural and/or functional independence that brain injury can selectively affect one case/font while leaving the others intact.

Some studies have raised the issue that letter shape may not necessarily be specified at the allographic level but at the level detailing the component strokes of a given letter. For example, Rapp and Caramazza [20] described individuals who produced letter replacement errors attributed to a deficit at the allographic level. An analysis of target-error characteristics revealed that they shared abstract motor features rather than visuospatial characteristics (see also [1,14,26,27]). Rapp and Caramazza [20] concluded that there is no obvi-

ISSN 0953-4180/12/$27.50 © 2012 – IOS Press and the authors. All rights reserved

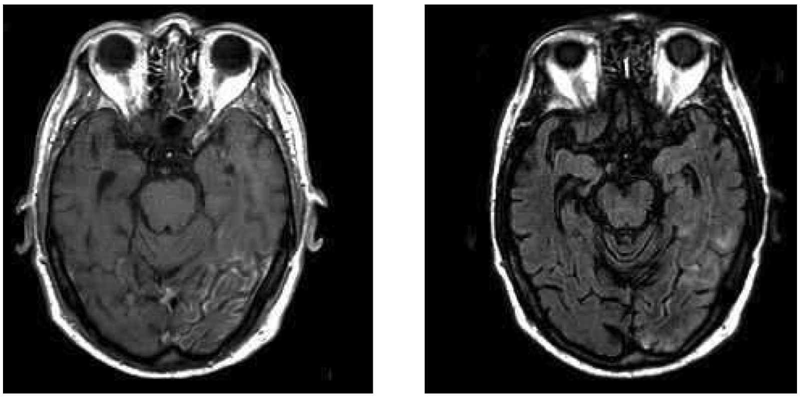

Fig. 1. MRI coronal sections showing left occipital and temporal (pole and inferior gyrus) hyperintensity with mild perilesional enhancement. Right side of the diagram correspond to the left side of the brain.

ous reason to assume that visuo-spatially based letter shape/representation is needed for writing or even recognition of letters. They offered an alternative interpretation according to which letter recognition and production are supported by abstract motorically-based representations.

Furthermore, Menichelli et al. [18] described the case of a patient, MN, who exhibited a severe impairment only in writing letters and words in upper-case print in the face of accurate production of the same stimuli in lower-case cursive. MN exhibited a generalized imagery deficits for both letter symbol formats. Thus her generalized imagery deficit did not impair her written production of lower case cursive. This finding led the authors to suggest that letter-shape production may not require explicit access to information about the visual attributes of letter shapes. Further evidence for this hypothesis may come from the present study.

This study documents the case of an individual, PS, who, after damage to the left temporal-occipital lobe due to acute encephalitis, showed a selective deficit in writing cursive-case single letters on dictation in face of the ability to write whole words using the impaired case allographs and to write upper-case print and words. This impairment, never observed before provides further support in favour of the independence of neural representations of font-specific letter types. More importantly, it highlights a unique dissociation between written production of single letters and that of individual letters within a word.

### *1.1. Case report*

P.S. is a 68 year-old, self-employed businessman, right-handed, with 11 years of formal education and good premorbid writing abilities. He suffered an acute encephalitis with no identified casual agent. He was admitted to the Neurological Unit of the "Ospedali Riuniti di Trieste" after the onset of the disease, showing headache, severe sensory transcortical aphasia, right hemiparesis, right hemianopia and mild fever. Complete neuropsychological evaluation on admission also demonstrated pure alexia, surface dysgraphia, associative visual agnosia, anterograde amnesia, attentional and executive deficits.

CT scan, serum PCR and blood cell count were normal. Cerebrospinal fluid analysis on admittance showed a moderate increase in protein levels and no cells; all microbiological analysis and paraneoplastic markers were negative. MRI imaging showed a FLAIR and T2 left occipital and temporal (pole and inferior gyrus) hyperintensity with mild perilesional Gd-T1 enhancement (Fig. 1).

After two weeks of combined high-dose i.v. steroid and antiviral treatment, MRI scan was unchanged, while perfusion SPECT documented moderate left occipital and temporal hypoperfusion (Fig. 2).

The patient's neurological and neuropsychological profile quickly improved: at the time of the first experimental testing he had fully recovered from right hemiparesis, but he still showed right hemianopia, language deficits (severe amnesic aphasia and dysgraphia), digit span reduction and deficits in visual recognition and image generation, while the attentional, executive and memory deficits almost completely recovered, although he was very slow in all the visual searching tasks.

P.S. could read target words only through the slow and inefficient letter-by-letter procedure. His performance in writing words and non-words on dictation taken from ENPA [2] was characterized by a regularity

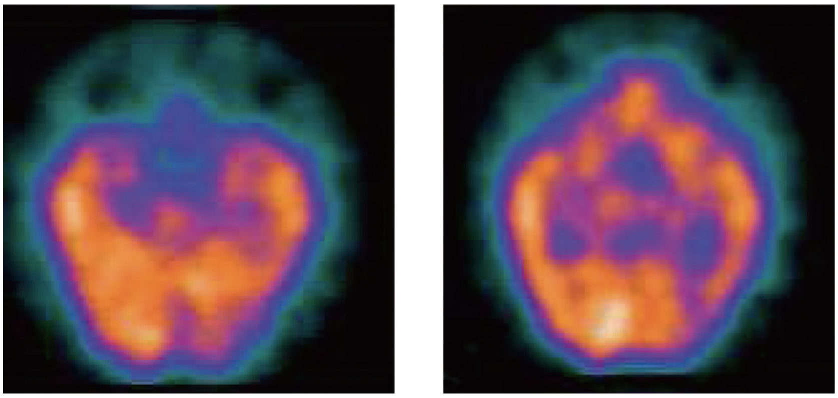

Fig. 2. SPECT documented moderate left occipital and temporal hypoperfusion.

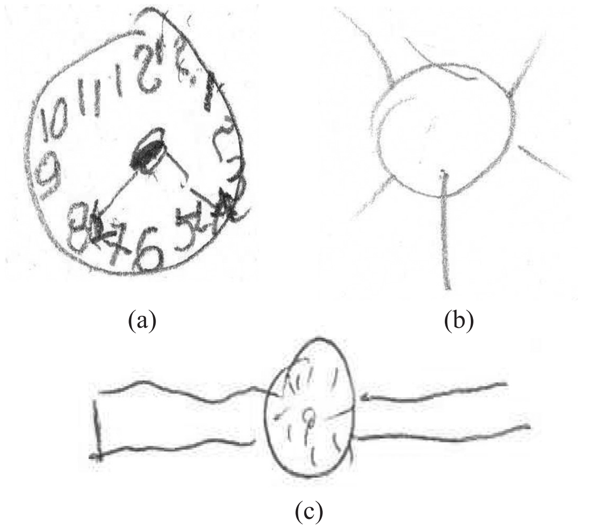

Fig. 3. PS's deficit in visual imagery for objects at $T_1$ fully recovered at $T_2$ as can be seen in his copy of a clock at $T_1$ (a) and in his drawing from memory of the same object at $T_1$ (b) and $T_2$ (c).

effect and by the production of phonologically plausible errors (e.g., "cuoco" written as "quoco"*: this non-word is pronounced exactly like "cuoco"). P.S. was able to write words both in lower-case cursive and in upper-case print.

P.S.'s performance was error-free in figure/ground discrimination (Screening Test from Visual Objects and Space Perception Battery, VOSP [25]) and on tasks requiring to match line-drawings of a) real objects (Minimal Feature View Task from Birmingham Object Recognition Battery, BORB [21]); b) objects observed from different perspectives (Foreshortened Matching Test from BORB). He could not draw from memory the same figures he was able to copy (see Fig. 3). For example, he correctly copied a triangle but when he was requested to draw the same shape he produced a square, despite being able to indicate the triangle between three different alternatives. He was poor in evocating the gesture associated with an object he could see but not manipulate (4/10 correct), better when requested to pantomime the use of named objects (8/10 correct), and he performed flawlessly when he could use real objects (10/10 correct). On the other hand, his performance was impaired in tasks requiring to discriminate real from unreal object line drawings (21/32 correct on Easy Object Decision from BORB) or to associate visual stimuli according to their semantic properties (18/30 correct on Association matching subtest from BORB). In addition, his performance was rather good (18/20 correct) with a subset of orally presented words taken from the Pyramids and Palm Trees Test [13], but he was impaired with the pictorial form of the same test (10/20 correct).[1] These findings seem to suggest that P.S. had a deficit in accessing the visual memory store from both visual perceptual analysis (i.e., in recognizing what an objects is) and the semantic system (i.e., in retrieving the mental visual image of an object in response to its name).

During our evaluation we noted that P.S. had severe difficulty in writing single letters in lower- and upper-case cursive, though he was able to write the same letters in upper-case print and he exhibited accurate production of lower-case cursive in writing words and non-words. Interestingly, the patient did not voluntarily adopt the compensatory strategy of spelling words in lower- or upper case cursive when trying to write single

[1] A group of normal subjects ($N = 10$), matched to the patient for age [age mean (range) = 67.8 (66–70) years] and with the same years of education [education = 11 years], was tested on the same subset of items reported here. All subjects scored 20/20 correct in both versions of the test.

letters. This behaviour persisted even when the patient was explicitly instructed to use this strategy. Thus, for example, when he was asked to write the letter "r", he was unable to produce it, either producing an unrecognisable scribble or saying he could not remember the letter. If suggested to write "r" as in the word "rana" (frog), what he did was to write the whole word "rana" and then copy the first letter. But he still could not carry out the task without writing the whole word. Notably, he was able to write arabic numbers up to 4-digits fairly well: there were some lexical errors but all digits were well formed.

This unusual pattern of performance, a deficit in allographic processing of a previously unreported nature, was thus the focus of our experimental investigation.

The investigation took place from December 2007 ($T_1$) to February 2008 ($T_2$). During this time, P.S.'s language and cognitive profiles still improved: on $T_2$ P.S. exhibited no other deficit then letter-by-letter dyslexia and the allographic deficit, improved but was still present.

Table 1 shows patient's performance on the general neuropsychological assessment at $T_1$ and $T_2$.

## 2. Experimental investigation

The patient underwent an extensive examination with the aim of shedding light on the nature of his writing deficit.

A group of normal subjects ($N = 10$), matched to the patient for age [age mean (range) = 67.8 (66–70) years] and education [all had 11 years of formal education], was tested on all the experimental tasks reported here, except those where P.S.'s performance was flawless. It should be reminded here that, for Italians, cursive is generally the default font. Our control participants were preliminarily asked to write seven words and three sentences without specifying the font. Every participant spontaneously adopted cursive case.

*Analysis 1: Letter form recognition*

*Task 1: Single letter naming*

P.S. was asked to read the 21 letters of the Italian alphabet presented in upper-case print and upper- and lower-case cursive. His accuracy was 100% both at $T_1$ and $T_2$.

*Task 2: Cross-case letter matching*

In this task each letter of the Italian alphabet ($N = 21$) was first presented in upper-case print and had to be associated to one of two letters written in lower-case cursive: one was the target letter and one a visually similar letter (e.g., "A"→"a" / "o"). Then each letter was presented in lower-case cursive and had to be associated to one of two letters written in upper-case print.

At $T_1$ P.S. declared he was not able to do the transcoding tasks in both case/style, despite clear understanding of the request. In fact, after a few incorrect attempts, he was first asked to read each stimulus aloud and then to choose the two allographs of the same letter. The patient performed at ceiling after this suggestion. However, when he was not allowed to read aloud the stimuli, he performed both tasks randomly (23/42, 54.7%, correct). Instead, at $T_2$ he performed 38/42, 90.5%, correct (with upper-case print, the letter "G" was matched to "q" instead of "g"; with lower-case cursive, letter "q" was matched with "P", letter "g" with "P", and letter "u" with "N"). The control group performed 100% correct in this task.

*Analysis 2: The dysgraphic deficit*

Writing single letters, words and non-words in upper-case print and upper and lower-case cursive was tested in order to evaluate the full extent of P.S.'s dysgraphia.

*Task 3: Writing single letters to dictation*

On two separate occasions P.S. was asked to produce to dictation, both on $T_1$ and $T_2$, the 21 letters of the Italian alphabet presented randomly, under three conditions: upper-case print, upper-case cursive and lower-case cursive. Since writing in lower-case print is not a familiar task in the patient's culture, this was not tested.

At $T_1$ his accuracy with upper-case print was 21/21, 2/21 in upper-case cursive (where the only correct letters were "O" and "Q") and 0/21 in lower-case cursive, where he mostly produced ill-formed unrecognizable letters (McNemar test, binomial distribution, 2-tailed, $N = 21$; upper-case print vs lower-case cursive: $p < 0.0001$; upper-case print vs upper-case cursive: $p < 0.0001$; lower-case cursive vs upper-case cursive, $p =$ n.s.). In the case of cursive P.S. claimed that he forgot how to write the letter. He was each time invited to try nonetheless. When he complied, errors mostly consisted of ill-formed, unrecognizable scribbles, with some pattern of perseveration across items.

Table 1
P.S.'s results of the general neuropsychological evaluation performed at 4 ($T_1$) and 12 ($T_2$) weeks after the onset of the disease

| Tasks | December 2007 ($T_1$) raw score (adjusted score or z score) | Febbrary 2008 ($T_2$) raw score (adjusted score or z score) |
|---|---|---|
| AAT-Spontaneous speech (0–5) | 5 5 5 **4** 5 5 | 5 5 5 5 5 5 |
| AAT-Token Test -errors (0–50) | **31** | 3 |
| AAT-Comprehension (0–120) | **46** | 106 |
| • Spoken-to Visual Matching (0–60) | **46** | 58 |
| • Written-to Visual Matching (0–60) | **0** | **48** |
| AAT-Oral Naming on visual confrontation (0–120) | **24** | 101 |
| AAT-Repetition (0–150) | **130** | 148 |
| AAT-Written language (0–90) | **39** | 82 |
| • Reading (0–30) | **15** | **23** |
| • Spelling with letter cards (0–30) | **0** | 30 |
| • Handwriting (0–30) | **24** | 29 |
| NPC-Reading arabic numbers (0–18) | **12** | 18 |
| NPC-Writing arabic numbers (0–18) | **15** | 18 |
| ENPA-Semantic fluency (animals) | **4** (**3.2**) | 12 (11.8) |
| Letter fluency (F A S) | **4** (**7.2**) | 20 (23.2) |
| Digit span fwd (0–8) | **3** (**2.75**) | 5 (4.75) |
| Digit span bwd (0–8) | **2** | **3** |
| Corsi span (0–8) | 5 (5) | 5 (5) |
| Words list | | |
| • immediate free recall (I-V trial) (0–80) | n.a. | 28 (29.2) |
| • 15' delayed free recall (0–16) | n.a. | **3** (**3.4**) |
| • 15' delayed yes/no recognition (0–32) | n.a. | 26 (25.7) |
| Prose Memory | | |
| • immediate recall (0–28) | 3 | 10 |
| • delayed free recall (0–28) | 2 | 8 |
| • total recall (0–28) | **2.5** (**3.5**) | 9 (10) |
| Warrington Faces (0–25) | 21 ($z = -0.95$) | 22 ($z = -0.42$) |
| P&P- pictures (0–20) | **10** | 18 |
| P&P- words (0–20) | 18 | 19 |
| BORB- Visual Association Matching Task (0–30) | **18** | 24 |
| Attentional Matrixes (0–60) | **30** (**26.25**) | 40 (46.25) |
| TMT- A (seconds) | **197"** (**195"**) | 78" (76") |
| TMT-B (seconds) | p.u. | **485"** (**417"**) |
| Stroop Test | | |
| • color naming | **14** (**19.6**) | 30 (38.6) |
| • word-color naming | **7** (**11.6**) | 13 (20.8) |
| MWCST | | |
| • number of categories (0–6) | p.u. | 3 |
| • perseverative errors | p.u. | 6 (5.75) |
| VOSP -Screening test (0–20) | 20 | n.a. |
| VOSP -Object Decision (0–20) | 15 | 15 |
| BORB- Minimal Feature View Task (0–25) | 25 | n.a. |
| BORB-Foreshortened Matching Test (0–25) | 25 | 25 |
| BORB-Object Decision A: easy (0–32) | **21** | 29 |
| IA (0–14) | 14 | 14 |
| IMA (0–72) | **49** | 62 |
| BORB-Drawing on verbal request (0–4) | **1** | 4 |
| BORB-Copy drawings (0–4) | 4 | 4 |

Note: data in boldface indicate performance outside the control range. Abbreviations used in the neuropsychological assessment: AAT-Aaachener Aphasie Test, Italian norms (Luzzatti, Willmes, De Bleser. Firenze, Organizzazioni Speciali, 1996); ENPA-Esame neuropsicologico per l'Afasia (Capasso & Miceli, 2001); FAS-Letter Fluency (Carlesimo, Caltagirone, & Gainotti, 1996); NPC-Number Processing and Calculation battery (Delazer, Girelli, Granà & Domahs, 2003); Span fwd-digit span forward (Orsini et al., 1987); Span bwd-digit span backward; Corsi test, spatial short-term memory (Spinnler & Tognoni, 1987); Words list-Semantically unrelated words list (Mauri et al., 1997); Prose Memory (Novelli et al., 1987); Warrington Faces-Short Recognition Memory for Faces (Warrington, 1996); BORB- Birmingham Object Recognition Battery (Riddoch & Humphreys, 1987); VOSP-Visual Object and Space Perception Battery (Warrington & James, 1991); P&P-Palm & Pyramid Test (Howard & Patterson, 1992); TMT-Trail Making Test (Giovagnoli et al., 1996); IMA-Ideomotor Apraxia (DeRenzi, Motti, & Nichelli, 1980); IA-Ideational Apraxia (De Renzi & Lucchelli, 1988); MWCST-Modified Wisconsin Card Sorting Test (Nelson, 1976; Caffarra et al., 2004); n.a. – task not administered; p.u. – patient unable to perform the task.

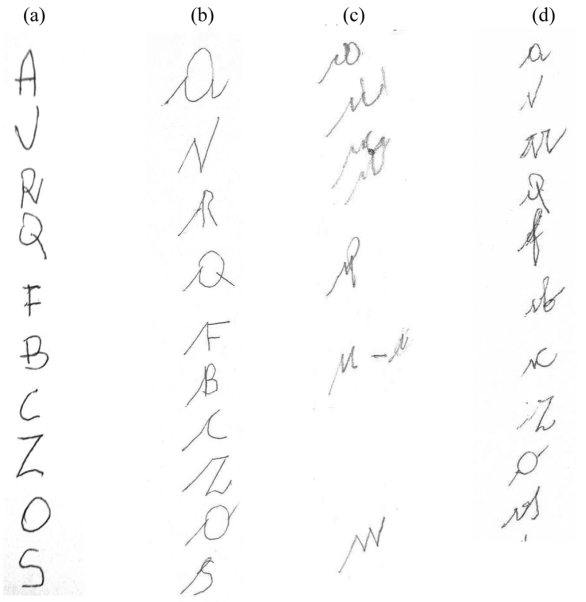

Fig. 4. Examples of PS's writing to dictation of different allographs at $T_1$ and $T_2$; letters A, V, R, Q, F, B, C, Z, O and S in upper-case print (a) and upper-case cursive at $T_1$(b), where only letters "A" and "O" were well-formed, and lower-case cursive at $T_1$ (c), where letters "f" and "o" where omitted, and $T_2$ (d).

At $T_2$ his performance improved: he accurately wrote 21/21 letters in upper-case print, 17/21 in upper-case cursive and 8/21 in lower-case cursive (McNemar test, binomial distribution, 2-tailed, $N = 21$; upper-case print vs lower-case cursive: $p < 0.005$; upper-case print vs upper-case cursive: $p =$ n.s; lower-case cursive vs upper-case cursive, $p < 0.0001$). In upper-case cursive the patient produced one ill-formed letter and 3 letters written in upper-case print; in lower-case cursive 10 letters were ill-formed ("b, c, h, i, l, m, n, p, r, s"; letter "u" was omitted; two were ill-formed upper-case print letters (G, Q) (Fig. 4). At $T_2$ errors were more similar to target letters than at $T_1$. Yet they were still ill-formed (especially when compared to his production on direct copy and in word dictation), and affected by perseveration across items.

All the control subjects were 100% correct in these tasks.

*Task 4: Direct copy of single letters*

In order to assess whether P.S.'s problem in writing in cursive involved the actual motor execution of letters (thus indicating a problem at the grapho-motor level or beyond), he was asked to copy the 21 letters of the Italian alphabet presented randomly, on two conditions: upper-case print and lower-case cursive.

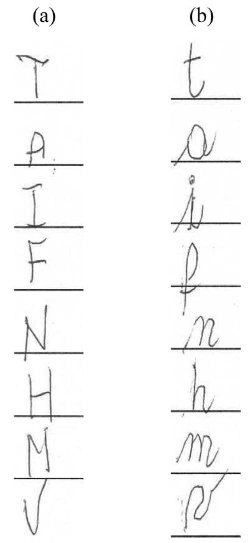

Fig. 5. Example of direct copy of upper- (a) and lower-case cursive (b) letters "t, a, i, f, n, h, m, v" at $T_1$.

At both $T_1$ and $T_2$ his performance was perfect (21/21), both in upper-case print and lower-case cursive (Fig. 5).

*Task 5: Delayed copy of single letters*

P.S. was requested to perform delayed copy of the 21 letters of the Italian alphabet presented in a random order in upper-case print and lower-case cursive. Each letter was presented for 5 seconds and then removed from view after which he was asked to reproduce it.

At $T_1$ his performance was perfect in upper-case print (21/21) but his accuracy in lower-case cursive was only 3/21 (the vast majority of his errors were ill-formed letters, the only letters written accurately were "a", "o" and "z") (McNemar test, binomial distribution, 2-tailed, $N = 21$; upper-case print vs lower-case cursive: $p < 0.0001$). At $T_2$ his performance in upper-case print was again perfect (21/21) and in lower-case cursive his accuracy was 16/21 (two letters were ill-formed →"g" and "r"; two letters were written in lower-case print → F, D; one letter was written in upper-case print → "L") (McNemar test, binomial distribution, 2-tailed, $N = 21$; upper-case print vs lower-case cursive: $p < 0.06$). (Figs 6 and 7). The performance of the control group was flawless in both tasks (upper-case print: 99.5% correct; lower-case cursive: 100%).

Thus P.S.'s performance was still better for upper-case print then lower-case cursive, and, although the difference was not significant, his performance was

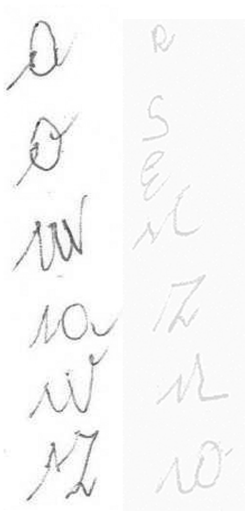

Fig. 6. Example of delayed copy of lower-cursive letters at $T_1$; letters: "a, o, m, u, v, r, r, s, e, q, z, l, i".

significantly lower than that of the control subjects (Bayesian $p$ (2-tailed) $< 0.000001$).[2]

*Task 6: Cross-case transcription of single letters*

P.S. was requested to transpose single letters from upper-case print to lower-case cursive and vice versa.

At $T_1$ he simply could not perform the task of converting upper-case print to lower-case cursive, despite several demonstrations by the examiner (0/21 correct). In contrast, he had no difficulties with transcribing from lower-case cursive to upper-case print (he only omitted letter "q") (McNemar test, binomial distribution, 2-tailed, $N = 21$; upper-case print vs lower-case cursive: $p < 0.0001$). At $T_2$ his performance was again almost perfect (20/21 correct) in the transcription of lower-case cursive to upper-case print (only "q" became "G"), while instead his accuracy was 15/21 in transcoding letters from upper-case print to lower-case cursive (two letters were ill-formed → r, v; three were written in lower-case print → f, h, p; the letter "e" was omitted) (McNemar test, binomial distribution, 2-tailed, $N = 21$; upper-case print vs lower-case cursive: $p < 0.06$). Control group performed 100% correct in both tasks. Thus P.S.'s performance was still better for upper-case print then lower-case cursive, and, although the difference did not reach the significance, his performance was significantly lower then that of the control subjects (Bayesian $p$ (2-tailed) $< 0.0000001$).

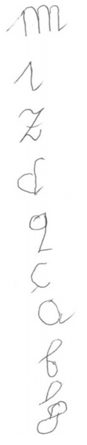

Fig. 7. Example of delayed copy of lower-cursive letters at $T_2$; letters: "m, i, z, d, q, c, a, b, g".

*Task 7: Writing words and non-words to dictation*

P.S. was asked to write to dictation 32 common words (16 high frequency and 16 low frequency, 5–8 letters) and 12 legal pseudo-words (5–8 letters) with one sound-to-one letter correspondence. The same stimuli were administered in two separate sessions in upper-case print and in lower-case cursive.

At $T_1$ P.S. correctly wrote 38/44 stimuli (28/32 words and 10/12 non-words) in lower-case cursive and 42/44 stimuli (31/32 words and 11/12 non-words) in upper-case print (McNemar test, binomial distribution, 2-tailed, $N = 44$; upper-case print vs lower-case cursive: $p =$ n.s.). There were no lexicality, frequency or length effects; the few errors consisted of letter omissions and substitutions (e.g.: coltello [knife]→written as "coltetto"). Importantly, there were no allographic errors: all letters in lower-case cursive were well-formed; sometimes P.S. produced the first letter of the target in upper-case cursive and then reverted the target to lower-case cursive (Fig. 8). At $T_2$ P.S.'s performance was almost perfect and did not differ from that of the control group, both in lower-case cursive (P.S.: 42/44, correct; mean of the controls: 43.2 correct, SD $= 1.03$; Bayesian $p$ (2-tailed) $=$ n.s.) and in upper-case print (P.S.: 43/44 correct; normal subject: 43.6 correct, SD $= 0.7$; Bayesian $p$ (2-tailed) $=$ n.s.).

*Task 8: Writing proper names to dictation*

P.S. was asked to write to dictation 20 names of persons and cities beginning with each letter of the

[2] P.S.'s performance was compared to that of the control sample ($N = 10$) with the program SingleBayes.exe [4]. This program uses Bayesian Monte Carlo inferential methods to test whether a patient's score is sufficiently below the scores of controls.

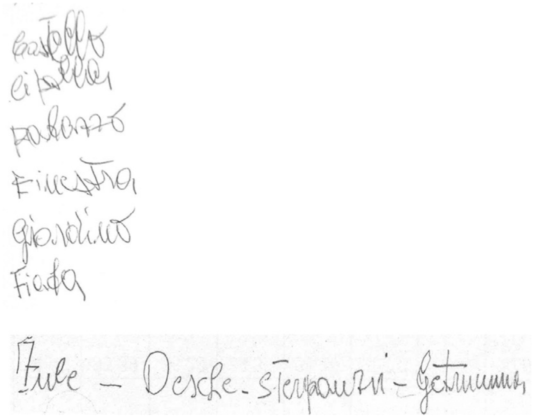

Fig. 8. Writing to dictation of words ("castello (castle), cipolla (onion), palazzo (palace), finestra (window), giardino (garden), fiala (ampoule)" and non-words ("fule, descia, sterpanzi, getrunna") in lower-case cursive at $T_1$.

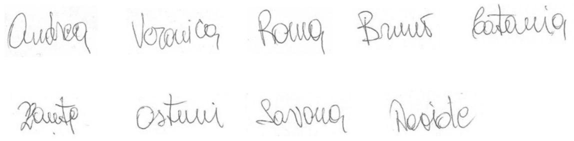

Fig. 9. Writing to dictation of proper names "Andrea, Veronica, Roma, Bruno, Catania, Zante, Ostuni, Savona, Davide" at $T_1$: all upper-case letters are well-formed.

Italian alphabet but "h" on two conditions: upper-case print and lower-case cursive. His accuracy was 20/20 both at $T_1$ and $T_2$ (Fig. 9).

*Task 9: Spelling words and non-words with mobile letters*

For this task P.S. was orally presented with the same list of words and pseudo-words used in Task 7 (handwriting to dictation). He was asked to spell each stimulus using sets of letter card provided: one set contained the letters of the alphabet printed in lower-case cursive letters and the other in upper-case print. In the first session he had to use the set in upper-case print, in the second he had to use the set in lower-case cursive.

P.S.'s performance on this task at $T_1$ was impaired, probably due to the combined effects of his verbal span deficit, his right visual field defect and his letter-by-letter dyslexia. He showed difficulties in finding the letters he needed, a marked word-length effect and difficulties in correcting his own mistakes even when he was aware of them. He frequently asked the examiner to repeat the word after choosing a few letters and he quite often gave up the task before completing the word. For these reasons, the task procedure was changed. PS was allowed to listen one or more times the target word, and only a subset of stimuli of the previous list was used (20 words and 8 non-words). Under this condition, his performance was fairly good and, more importantly, it was similar regardless of the case or font of letters (upper-case print: 24/28, lower-case cursive: 21/28 (McNemar test, binomial distribution, 2-tailed, $N = 28$, $p =$ n.s.). The errors consisted of letter substitutions, omissions and insertions. P.S. had no problem with this task at $T_2$, in fact he performed 100% correct as the control group.

*Task 10: Form cueing in written word completion*

Unfortunately, this task was administered only at $T_2$. P.S. was visually presented with a list of 20 words and 20 non-words, written in lower-case cursive, missing the initial letter [every target started with a different letter of the Italian alphabet except letter "h"; e.g., _arca/barca (boat); _ita/bita (non-word)]; his task was to complete the word stimulus in the case in which it was presented and he was told what the target word was.

P.S. correctly completed most of the words (18/20 correct) and non-words (17/20 correct). The few errors were on letter "q" (in one case it was omitted and in the other case it was written in upper-case print), on letter "f" (substituted by letter "l" in a word (e.g., fido→lido) and on letter "z" in a non-word (finafo→zinafo); letter "p" was written in lower-case print in a non-word. Control group performed 396/400 (99%) correct on this task.

### 2.1. *Summary and discussion for Analysis 1 & 2*

Table 2 summarizes P.S.'s performance on Tasks 1–10 at $T_1$ and $T_2$.

The results reported hereafter indicate that P.S. had a striking difficulty in producing, but not in recognizing, single letters in cursive, both in upper- and lower-case. This deficit persisted for two months,although improving over time. P.S.'s performance on tasks 3–6 document this dissociation: he suffered from damage at levels prior to the stages involved in the written production of letter shapes. The fact that the direct copy of lower-case cursive is intact, indicates that the difficulty in lower-case production is not purely motoric. These results localize his difficulties in lower-case cursive letter production at the allographic stage.

Tasks 7–8 showed that P.S.'s difficulties in writing lower-case cursive were associated only with single letters as he was able to write the same letters in words and

Table 2
Summary of P.S.'s performance (% correct) with upper-case print versus lower-case cursive at $T_1$ and $T_2$

| Tasks | Print (%) | | Cursive (%) | | Control mean (%) |
|---|---|---|---|---|---|
| | $T_1$ | $T_2$ | $T_1$ | $T_2$ | |
| Writing single letters to dictation | 100 | 100 | 0 | 38 | 100 |
| Direct copy of letters | 100 | 100 | 100 | 100 | n.a. |
| Delayed copy of letters | 100 | 14 | 100 | 76 | 100 |
| Copy transcoding single letters (a→A; A→a) | 95 | 0 | 95 | 71 | 100 |
| Single letter naming | 100 | 100 | 100 | 100 | n.a. |
| Cross case matching of single letters | p.u. | 95 | p.u. | 86 | 100 |
| Writing words and non-words to dictation | 95 | 86 | 98 | 95 | 98 (A), 97 (a) |
| Spelling with mobile letters | 86 | 100 | 75 | 100 | 100 |
| Words and non-words completion | n.a. | n.a. | n.a. | 87 | 99 |

Note: n.a. – task not administered; p.u. – patient unable to perform the task; A-upper case print; a- lower case cursive.

non-words. Moreover, he wrote fluently proper names and he correctly used capital letters when needed.

P.S.'s errors in lower- and upper-case cursive consisted of ill-formed letters and, to a lesser extent, production of different allographs. Task 9 provided further evidence that there was a problem with retrieving the single letter form from imagery. When given the right letter form, P.S. was able to choose it appropriately. Task 10 seemed to show that retrieving a whole word representation was enough to overcome his single letter retrieval deficit in lower cursive.

*Analysis 3: The accessibility of cursive representations*

Writing words and non-words to dictation and writing proper names on dictation suggested at least some access to upper- and lower-case cursive letter forms. In the following tasks P.S.'s ability to explicitly generate and inspect mental images of allographic forms was examined.

*Task 11: Form judgement*

P.S. was requested, as in Venneri et al. [24], to judge which one out of three letters orally provided by the examiner (e.g. "O, Q, I" or "m, n, f") was visually dissimilar to the other two (P.S.'s own writing was checked in order to build this task).

At $T_1$, he could not carry out this task in any format. At this time P.S. was also unable to perform a similar task with 10 triplets of objects (e.g., wheel, ball and stick). His difficulties in these tasks were thus due to a general impairment in visual imagery, as already shown in the neuropsychological assessment. At $T_2$ his accuracy was 3/10 with lower-case cursive allographs but 10/10 with upper-case print forms (upper-case print vs lower-case cursive: Mann-Whitney test, $z = -2.8$, $p$ (2-tailed) $< 0.005$) and with objects. All the control subjects performed these three tasks accurately.

*Tasks 12–13: Association of lower-cursive letters to different frames and pointing to a letter stroke in upper-case print*

The following tasks, originally devised by Lambert et al. [14], were administered only at $T_2$ to further assess P.S.'s knowledge of the general shape of a target letter (at the allographic level). The general principle of the tasks involved pointing to visual characteristics in a multiple-choice questionnaire (one question for each letter of the Italian alphabet), without requiring written production of the letter or its verbal description.

For lower-case cursive letters, questions involved the relative size and position of a letter on a baseline, represented by frames (see Fig. 10) corresponding to the different types of cursive letters: corpus-sized letters ("a", "c", "e", etc.), letters with an upward stroke ("d", "l", "t", etc.), a downward strokes ("g", "q", "p") and upward and downward stroke ("f"); P.S.'s task was to point the frame in which he could write the letter orally provided by the examiner in lower-case cursive style. As for upper-case print letters, questions concerned the direction of the strokes of a target letter. The multiple choices comprised 4 strokes with different forms (straight, curves) and orientations (vertical, horizontal, oblique) (see Fig. 11). P.S. was asked to imagine each letter of the Italian alphabet, spoken by the examiner, in upper-case print and then to point the stroke which is present in the letter.

P.S. performed 11/21 (52.4%) correct in lower-case cursive and 19/21 (90.5%) correct in upper-case print (upper-case print vs lower-case cursive: Mann-Whitney test, $z = -2.7$, $p$ (2-tailed) $< 0.01$). He was further requested to perform the same tasks with visually presented letters in both formats. His accuracy was 18/21 (85.7%) with lower-case cursive and 21/21 with upper-case print (upper-case print vs lower-case cursive: Mann-Whitney test, $z = -1.8$, $p$ (2-tailed) $=$ n.s.).

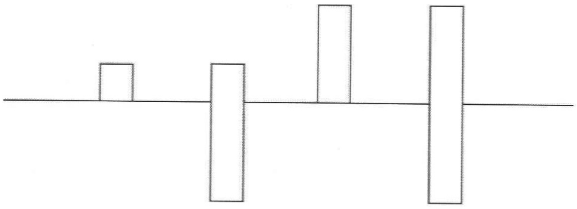

Fig. 10. Mental imagery of lower-cursive letters task.

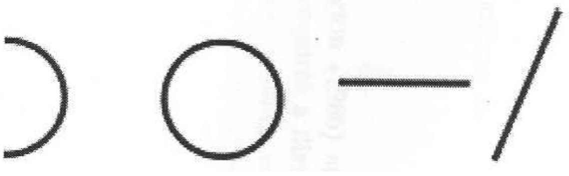

Fig. 11. Mental imagery of upper-case print letters task.

On the imagery tasks, control subjects' accuracy was 209/210 (99.5%) and 182/210 (86.7%) in lower-case cursive and in upper-case print respectively (lower-case cursive imagery task, P.S.: 11/21, correct; mean of the controls: 20.9 correct, SD = 0.3; Bayesian $p$ (2-tailed) $< 0.0000001$; upper-case print, P.S.: 19/21, correct; mean of the controls: 18.2 correct, SD = 2.1; Bayesian $p$ (2-tailed) = n.s.). In the control tasks, normal subjects performed 209/210 (99.5%) correct with lower-case cursive and 195/210 (92.8%) correct with upper-case print.

*Task 14: Oral description of letter strokes*

PS was requested to provide, from his own imagination, an oral description of letter strokes across two conditions: lower-case cursive and upper-case print. He was instructed about the different possible types of stroke: straight/curve and vertical/horizontal/oblique.

His accuracy in lower-case cursive was 13/21 (61.9%), and his answers often described upper case print instead of lower cursive: "q" had a curve and an oblique stroke → Q; with "f" and "h" he did not mention the curve stroke; he didn't know which is the "s" shape; "l" had a vertical and horizontal stroke → L; "m" and "n" didn't have curve strokes but only vertical and oblique strokes→ M, N). When administered the same task with visual presentation of letters, his accuracy was 21/21. In upper-case print he obtained a 21/21 accuracy both on the mental imagery and the visual presentation conditions.

The control group performed only the imagery tasks. They were 190/210 (90.4%) correct with lower-case cursive and 192/210 (91.4%) correct with upper-case print.

### 2.2. Summary Analysis 3

These findings suggest a general impairment in generating visual images of letters. At $T_1$ this deficit generalised also to objects shapes, despite evidence of understanding the task. At $T_2$, however, the impairment affected only letter shapes. When P.S. was requested to access single letter shapes in lower cursive, he recovered the correspondent allograph in upper-case print instead. A dissociation thus emerged between his ability to explicitly generate and inspect mental images of objects and of allographic upper-case print forms (almost perfect) in contrast to lower-case cursive forms (impaired).

## 3. Conclusions

The main feature of this case was a severe impairment in writing single letters in lower- and upper-case cursive. In contrast the patient showed: a) perfect recognition of the same allographs he could not produce, b) accurate production of single letters in upper-case print, c) accurate production of lower-case cursive in writing words and pseudo-words, d) spontaneous production of accurate upper-case cursive allographs in writing the initial letter of common words and proper names to dictation.

Within this pattern, P.S. first displayed a general impairment in mental imagery regarding both letters and objects. After partial recovery, however, a dissociation emerged between his ability to explicitly generate and inspect mental images of objects and of allographic upper-case print forms (almost perfect) in contrast to lower-case cursive forms (impaired).

This case contributes to our understanding of letter-shape representation in written language production. It provides strong support for previous reports indicating the neural independence of different types of case and font-specific letter-shape information at an allographic level. In particular it shows the reverse pattern with respect to the case reported by Menichelli et al. [18], whereby upper-case print was impaired in contrast to preserved lower-case cursive.

Unlike patient M.N., but similarly to other patients as, for instance, the patient reported by Venneri et al. [24], P.S.'s difficulty in generating and inspecting mental images of allographs was, at least at $T_2$, letter-specific and restricted to the font-case he could not produce. This clearly rules out the possibility that P.S.'s difficulty could arise from problems understanding instructions.

A deficit in generating and inspecting mental images of allographs they cannot produce is thus a common association in patients with allographic disorders. Importantly, the present case seems to provide further evidence that letter-shape production does not require *explicit* access to information about the visual attributes of letter shapes. In our opinion this conclusion is strongly supported by the contrast found in P.S.'s performance between writing single letters and writing whole words. Retrieval of single letters on specific request conceivably requires explicit access to information about a letter shape. In writing a whole word, or even a non-word, accessing letter shapes may be a more automatic process once the sequence in the graphemic output buffer is activated. Indeed what seems to trigger the letter sequence automatically is not the orthographic representation in the lexicon but rather the representation built in the graphemic output buffer. This would explain why no difference was found between words and legal pseudo-words. A lexical effect could have been advocated to explain P.S.'s performance were pseudo-words used for this investigation graphically similar to real words, e.g., obtained by changing one or two letters. Non-words obtained by changing one or two letters might have been assimilated to real words, hence a facilitation effect extending to non-words. However, most non-words in our experimental material, though legal sequences of letters matched in length to the real words we used, bore no resemblance to any real word. Thus, it is likely that any abstract letter sequence made available in the graphemic output buffer implicitly activates cursive format-specific information in P.S. Completing a word with a single letter (as in task 10) might nonetheless require the activation of the whole letter sequence. In order to add a letter to a given letter sequence to write a real word, one might activate the whole word first in the lexicon and, if writing is required, then in the graphemic output buffer. Moreover, since the word to be completed is already written in the wanted format, this fact alone may possibly help the production of the remaining letter, again in a more automatic way with respect to the production of a single letter in isolation.

An alternative possible explanation for the dissociation featured by P.S. may require positing that independent format-specific representations exist for individual letters and for whole words. Storing format specific representations for all known words, however, would be a very uneconomic, given their high occurence in comparison to the very limited number of letter representations in a given language (21 in Italian). Indeed, some whole word format- specific representations may exist: words like "Coca Cola" are usually written in a specific format, which is recognized and "read" even by illiterate children (via right hemisphere mechanisms [6]). Other whole word format specific representations may exist as well, but in this case they may not be stored so solidly even in normal readers and writers. It is hard to tell even how their integrity could be tested. If, nonetheless, word format specific representations would actually exist, however, P.S.'s good performance with pseudo-words would still have to be explained.

Within a problem in generating a specific font or case, the allographic retrieval process for word-like letter sequences would be, thus, largely *implicit* and based on the inherent mechanisms of procedural rather than declarative memory. This proposal is consistent with the view put forward by case reports of dissociations between preserved production of letters and imagery deficits of the same letters [7,11,22] and by the case reported by Rapp and Caramazza [20] whereby allographic agraphia was influenced by graphomotor similarity and not by the letter shape. Use of implicit rather than explicit processes may more easily occur in lower-case cursive, where single letters in a word-like sequence are graphically connected, than in upper-case print, where each letter is not connected to the other letters. If this is true, single letter deficits like that reported here would be much more likely found in lower cursive than in upper print. As Menichelli et al. [18] also pointed out, however, is important to note that the separate question of whether even *implicit* access to the visual attributes of letters (and not just to motor patterns) is necessary for written letter production remains unanswered. The intricacies of the neurological network sustaining appropriate letter writing will be finally understood only when enough cases of neat dissociation like the present one are reported.
